# Supplementary material for: Multi‐dataset identification of innovative feature genes and molecular mechanisms in keratoconus
Source: J Cell Mol Med. 2024 Sep 19;28(18):e70079. doi: 10.1111/jcmm.70079 (PMC11412914; doi:10.1111/jcmm.70079)
Supplement: Supplementary file 2 — Material S2. [file JCMM-28-e70079-s002.zip › 7.GSEA analysis/6.SNORA74B/KEGG_GLYCOSPHINGOLIPID_BIOSYNTHESIS_LACTO_AND_NEOLACTO_SERIES.html]

Details for gene set KEGG\_GLYCOSPHINGOLIPID\_BIOSYNTHESIS\_LACTO\_AND\_NEOLACTO\_SERIES[GSEA]

|  || Dataset | SNORA74B.group.cls#h\_versus\_l |
| Phenotype | group.cls#h\_versus\_l |
| Upregulated in class | l |
| GeneSet | KEGG\_GLYCOSPHINGOLIPID\_BIOSYNTHESIS\_LACTO\_AND\_NEOLACTO\_SERIES |
| Enrichment Score (ES) | -0.4668544 |
| Normalized Enrichment Score (NES) | -1.3526404 |
| Nominal p-value | 0.09856263 |
| FDR q-value | 0.33024424 |
| FWER p-Value | 0.93 |
Table: GSEA Results Summary

  

Fig 1: Enrichment plot: KEGG\_GLYCOSPHINGOLIPID\_BIOSYNTHESIS\_LACTO\_AND\_NEOLACTO\_SERIES      
 Profile of the Running ES Score & Positions of GeneSet Members on the Rank Ordered List

  

| SYMBOL | TITLE | RANK IN GENE LIST | RANK METRIC SCORE | RUNNING ES | CORE ENRICHMENT || 1 | ABO | na | 2752 | 0.316 | -0.0208 | No |
| 2 | B3GALT2 | na | 4672 | 0.236 | -0.0325 | No |
| 3 | B3GALT5 | na | 5357 | 0.213 | -0.0128 | No |
| 4 | FUT7 | na | 5725 | 0.201 | 0.0138 | No |
| 5 | FUT2 | na | 6556 | 0.175 | 0.0222 | No |
| 6 | B3GNT3 | na | 9058 | 0.112 | -0.0291 | No |
| 7 | FUT3 | na | 9792 | 0.097 | -0.0323 | No |
| 8 | B4GALT2 | na | 10010 | 0.092 | -0.0215 | No |
| 9 | ST8SIA1 | na | 11894 | 0.062 | -0.0644 | No |
| 10 | FUT1 | na | 14378 | 0.032 | -0.1301 | No |
| 11 | FUT9 | na | 14949 | 0.025 | -0.1419 | No |
| 12 | FUT4 | na | 15265 | 0.022 | -0.1470 | No |
| 13 | FUT6 | na | 16325 | 0.011 | -0.1754 | No |
| 14 | B4GAT1 | na | 18322 | -0.010 | -0.2311 | No |
| 15 | B4GALT1 | na | 20837 | -0.038 | -0.2965 | No |
| 16 | ST3GAL4 | na | 24766 | -0.094 | -0.3923 | No |
| 17 | B4GALT4 | na | 24861 | -0.096 | -0.3773 | No |
| 18 | B3GNT5 | na | 25750 | -0.112 | -0.3822 | No |
| 19 | B4GALT3 | na | 27091 | -0.141 | -0.3947 | Yes |
| 20 | B3GNT2 | na | 27484 | -0.151 | -0.3781 | Yes |
| 21 | ST3GAL6 | na | 27779 | -0.159 | -0.3572 | Yes |
| 22 | GCNT2 | na | 29274 | -0.203 | -0.3628 | Yes |
| 23 | B3GNT4 | na | 29412 | -0.207 | -0.3285 | Yes |
| 24 | ST3GAL3 | na | 34214 | -0.585 | -0.3587 | Yes |
| 25 | FUT5 | na | 34557 | -0.731 | -0.2336 | Yes |
| 26 | B3GALT1 | na | 34723 | -1.290 | 0.0001 | Yes |
Table: GSEA details [plain text format]

  

Fig 2: KEGG\_GLYCOSPHINGOLIPID\_BIOSYNTHESIS\_LACTO\_AND\_NEOLACTO\_SERIES      
 Blue-Pink O' Gram in the Space of the Analyzed GeneSet

  

Fig 3: KEGG\_GLYCOSPHINGOLIPID\_BIOSYNTHESIS\_LACTO\_AND\_NEOLACTO\_SERIES: Random ES distribution      
 Gene set null distribution of ES for **KEGG\_GLYCOSPHINGOLIPID\_BIOSYNTHESIS\_LACTO\_AND\_NEOLACTO\_SERIES**
